# News Items

**Published:** 1885-03

**Authors:** 


### NEWS ITEMS.

General regret was felt by the medical profession on the announcement that the publication of the *Index Medicus* had been discontinued by the publishing house of its former publisher, F. Leypoldt, of New York.

Authoritative announcement is now made by its editors, Dr. John S. Billings and Dr. Robert Fletcher, that Mr. George S. Davis, of Detroit, Michigan, has undertaken to continue its publication on the same general plan as the preceding numbers.

THE NEW ST. LUKE'S HOSPITAL BUILDING.—Since the issue of the last number of the JOURNAL AND EXAMINER the new building for St. Luke's Hospital has been so far advanced that the patients have been transferred to it, and the hospital is in good working order.

The structure is not yet complete. The old buildings stand upon the ground which is to be used for the pavilion intended for the surgical wards and the ward designed for children. It is contemplated to remove the old buildings at an early date, and to proceed to erect the new pavilion, when the structure will present the form of a hollow square. The executive portion of the building extends east and west, whilst the pavilions are arranged to run north and south, so as to secure the greatest amount of exposure to sun-light in the wards and private rooms.

The heating and ventilation of the buildings are on the same plan as that of the John Hopkins Hospital. To avoid too high temperature from the steam heating, more radiating surface,

—in the direct radiation,—has been introduced, which, with the careful ventilation, gives an agreeable temperature in the building.

The accident ward and the dispensary are located on the ground floor, whilst the amphitheater, with the anæsthetizing and the recovery rooms, are at the top of the building, accessible by elevator. The amphitheater is remote from the wards and is placed where good light can be had.

In its construction and its internal arrangements, the hospital is fully abreast of the times.

The autopsy amphitheater—the wards for contagious diseases and the laundry are located in a detached building.

The authorities of the hospital have anticipated its continued growth and have secured additional ground adjoining, upon which an extension of the present building may be erected, when needed, which will be in keeping with the plan of the present structure.

---

The Illinois State Medical Society will meet this year in Springfield, on the third Tuesday of May, (19th).

---

The Executive Committee of the Sanitary Council of the Mississippi valley have, by direction of the Council, addressed a memorial to the President of the United States asking that the National Board of Health be convened and authorized to proceed to make such provision as may seem best, to secure the country against the threatened invasion of the cholera.

---

The death of William Braithwaite, M. D., the founder of the *Retrospect of Medicine*, occurred at his home in Leeds, England, on the 31st of January.

His son, Dr. James Braithwaite, will continue to publish the *Retrospect*, which was first begun in 1840.

Commencement of the College of Physicians and Surgeons.—The third Annual Commencement of the Chicago College of Physicians and Surgeons was held at the Grand Opera House on Tuesday afternoon, March 10th.

The President, Professor A. Reeves Jackson, M. D., conferred the degree of Doctor of Medicine upon sixty candidates.

Professor R. L. Rea has resigned the Chair of the Principles and Practice of Surgery. He will lecture, hereafter, upon the subject of Military Surgery.

